# Ferulic Acid as a Protective Antioxidant of Human Intestinal Epithelial Cells

**DOI:** 10.3390/antiox11081448

**Published:** 2022-07-26

**Authors:** Hye-Jeong Hwang, So Rok Lee, Ju-Gyeong Yoon, Hye-Ri Moon, Jingnan Zhang, Eunmi Park, Su-In Yoon, Jin Ah Cho

**Affiliations:** 1 Department of Agrofood Resources, National Institute of Agricultural Sciences, Rural Development Administration, Wanju 55365, Korea; hjh1027@korea.kr; 2 Department of Food and Nutrition, Chungnam National University, 99, Daehak-ro, Yuseong-gu, Daejeon 34134, Korea; sj807sr@cnu.ac.kr (S.R.L.); jgyoon@o.cnu.ac.kr (J.-G.Y.); mhyeri02108@o.cnu.ac.kr (H.-R.M.); lkl1997@o.cnu.ac.kr (J.Z.); 3 Department of Food and Nutrition, Hannam University, 1646, Yuseung-daero, Yusung-gu, Daejeon 34054, Korea; eunmi_park@hnu.kr; 4 Research Center for Microbiome-Brain Disorders, Chungnam University, Daejeon 34134, Korea; suinyoon@cnu.ac.kr

**Keywords:** ferulic acid, ER stress, pro-inflammatory response, intestinal epithelial cells

## Abstract

The intestinal epithelial barrier is the primary and most significant defense barrier against ingested toxins and pathogenic bacteria. When the intestinal epithelium barrier is breached, inflammatory response is triggered. GWAS data showed that endoplasmic reticulum (ER) stress markers are elevated in Inflammatory Bowel Disease (IBD) patients, which suggests ER stress regulation might alleviate IBD symptoms. Ferulic acid (FA) is a polyphenol that is abundant in plants and has antioxidant and anti-inflammatory properties, although it is unclear whether FA has these effects on the intestine. Therefore, we investigated the effect of FA in vitro and in vivo. It was found that FA suppressed ER stress, nitric oxide (NO) generation, and inflammation in polarized Caco-2 and T84 cells, indicating that the ER stress pathway was implicated in its anti-inflammatory activities. The permeability of polarized Caco-2 cells in the presence and absence of proinflammatory cytokines were decreased by FA, and MUC2 mRNA was overexpressed in the intestines of mice fed a high-fat diet (HFD) supplemented with FA. These results suggest that FA has a protective effect on intestinal tight junctions. In addition, mouse intestine organoids proliferated significantly more in the presence of FA. Our findings shed light on the molecular mechanism responsible for the antioxidant effects of FA and its protective benefits on the health of the digestive system.

## 1. Introduction

The gut epithelial barrier is the first and most important defense against ingested pathogens such as toxins, viruses, and bacteria. Innate immune response in gut epithelial cells is activated through the Toll-like receptor (TLR) pathway, the nucleotide-binding oligomerization domain (NOD) pathway, or autophagy, which are the characteristics of cell stress response. Furthermore, a fine balance exists between intestinal microbiota and the ability of the intestinal epithelial barrier to protect intestinal epithelium, which largely determines gut health. Thus, intestinal epithelial cells play an important role in immunological homeostasis.

Polyphenols are important antioxidants with antibacterial, anti-allergic, antiviral, and anti-cancer properties, and ameliorate the effects of immunological inflammatory diseases. Ferulic acid (FA, (2E)-3-(4-hydroxy-3-methoxyphenyl)prop-2-enoic acid) is a dietary polyphenol and is abundant in the cell walls of plants such as wheat, oats, coffee beans, apples, oranges, peanuts, pineapples, and artichokes [1]. Whole grains are a rich source of phenolic compounds, mainly hydroxycinnamic acids, which is FA [2,3,4]. In foods, FA exists mainly as the trans-isomer and is covalently bound to arabinoxylan chains of cell wall polysaccharides by ester bonds [5,6]. FA exhibits a wide range of biological activities but is best known for its ability to neutralize reactive oxygen species (ROS) [7,8]. However, recent studies have shown FA has diverse pharmacological effects such as anti-diabetic [8,9], and anti-bacterial effects against *E. coli*, *Helicobacter pylori*, and *Shigella sannei* [10], neuroprotective effects, especially in the context of amyloid β protein toxicity [11,12,13,14,15,16], a brightening effect on skin [17], and anti-cancer effects [18,19,20]. In addition, FA has been reported to induce hypoxia and enhance the angiogenesis of human umbilical vein endothelial cells (HUVEC) by increasing the expressions of HIF-1α and vascular endothelial growth factor (VEGF) [21] and to specifically enhance the neurogenic differentiations and survivals of different types of stem cells in vitro and in vivo [22,23]. Most interestingly, many studies have investigated the anti-inflammatory effects of FA on inflammatory mediators in rats [24] and its suppression of neuronal ER stress by regulating the unfolded protein response signaling pathway and apoptosis [25]. However, few studies have examined the mechanism responsible for the anti-inflammatory effect of FA on polarized human intestinal epithelial cell lines.

Cells act in a coordinated manner to maintain homeostasis in response to sudden changes in their environment [26]. Disruption of cellular physiologic equilibrium is called cell stress, and among them, disruptive events in ER, which is the organelle mainly responsible for protein synthesis and signal transduction, cause ER stress. When ER stress sensors on the ER membrane, such as inositol requiring enzyme 1α (IRE1α), protein kinase R (PKR)-like endoplasmic reticulum kinase (PERK), and ATF6, are activated by ER stress, BiP (a 78 kDa binding-immunoglobulin protein) bound to the ER luminal domain of ER stress sensors is released into ER lumen [27,28]. Of the three ER stress sensors, IRE1α is the most potent sensors among three and participates in the noble innate immune surveillance system [29] IRE1α is activated by autophosphorylation and this leads to endoribonuclease activity and the splicing of X-box binding protein 1 (XBP1) mRNA to produce the spliced form called XBP1s; is a potent transcriptional activator. XBP1s catalyzes the synthesis of the ER-chaperones such as BiP and proteins related to unfolded protein response (UPR) to relieve initial ER stress [27,30,31,32]. However, persistent ER stress leads to a signaling switch from survival pathways to apoptosis due to the sustained activation of PERK, which results in the activations of C/EBP homologous protein (CHOP) and DNA damage-inducible protein 34 (GADD34), the inhibition of eukaryotic initiation factor 2α (eIF2α) inducing apoptosis of the cells [33,34]. 

Recent genome-wide association studies (GWAS) have shown inflammatory bowel disease (IBD) is causally associated with ER stress and that chronic ER stress is a characteristic of various inflammatory diseases [35,36,37,38,39]. 

Therefore, we investigated the effect of FA on ER stress-associated inflammatory response of intestinal epithelium under physiologic and pathologic conditions in vitro and in vivo.

## 2. Materials and Methods

### 2.1. Reagents

FA, Thapsigargin, Hanks’ balanced salts (HBSS), 4-(2-hydroxyethyl)-1-piperazineethanesulfonic acid (HEPES), and 4 kDa fluorescein isothiocyanate-dextran (FITC-dextran) were purchased from Sigma Aldrich Co., (St. Louis, MO, USA). Lipopolysaccharide (LPS) was purchased from Invivogen (San Diego, CA, USA). IFNγ, TNFα, and IL1β were purchased from JW Creagene (Seongnam, Gyeonggi-do, Korea). FA was dissolved in 95% ethanol and Tg was dissolved in 100% DMSO and was used at a final concentration of 3 uM. Chloroform was purchased from JUNSEI (Junsei Chemical Co., Ltd., Tokyo, Japan) and isopropanol from DUKSAN (Reagent Duksan Co., Ansan, Gyeonggi-do, Korea).

### 2.2. Cell Culture

Human intestinal epithelial cells, Caco-2 and T84, were purchased from the American Type Culture Collection (Manassas, VA, USA). ER stress marker-knockout Mouse Embryonic Fibroblast (MEF) cells were a generous gift from David Ron (University of Cambridge, Cambridge, UK). The cells were cultured in Dulbecco’s Modified Eagle’s medium (DMEM; Thermo Fisher Scientific, Waltham, MA, USA) with 1% Penicillin-Streptomycin (Sigma-Aldrich Co., Saint louis, MO, USA) and 10% heat-inactivated fetal bovine serum (FBS) (Thermo Fisher Scientific) at 37 °C under an atmosphere containing 5% CO_2_. 

To polarize Caco-2 and T84 cells, rat tail collagen I (Corning Inc., New York, NY, USA) was diluted in 0.02 N acetic acid to a final concentration of 100 μg/cm^2^ on the polycarbonate membrane in the Transwell plates (Corning), and then dried overnight under a laminar flow in the cell culture hood. Residual acetic acid was removed by washing with PBS and cells were plated onto collagen coated Transwell membranes, cultured, and polarized for 2–3 weeks. Polarization represented by resistance of the monolayer of the cells was measured by volt meter (World Precision Instruments, Sarasota, FL, USA) before the experiments. Polarized cells were pretreated with FA apically for 24 h, then treated apically with cytokine cocktail (CT: 50 ng/mL human TNFα + 50 ng/mL human IFNγ + 25 ng/mL human IL1β + 10 μg/mL LPS) for additional 24 h.

### 2.3. Experimental Animals

#### 2.3.1. Mice

Male C57BL/6 mice aged 4 weeks were purchased from Daehan BioLink (Eumseong, Chungcheongbuk-do, Korea), housed in groups of five per cage and had free access to food and tap water in a pathogen-free animal care facility at RH 50 ± 5% and 20 ± 1 °C under a 12:12 h light–dark cycle (lights on at 8:00 a.m.). After one week of quarantine, mice were allocated to groups of 4 and fed a standard chow diet (S: #D12450J, Research Diet Inc., New Brunswick, NJ, USA), a standard chow diet with FA (SF: 50 mg/kg FA in chow diet), a high-fat diet (HF: #D12492, Research Diet Inc.), or a high-fat diet containing FA (HFF: 50 mg/kg FA in the HF) for 8 weeks. Animals were fasted for an hour before sacrifice. All animal experiments were approved beforehand by the Committee of Animal Care and Experiment of Hannam National University (HNU 2016-6) and were carried out in accordance with the requirements of the National Institutes of Health Guide for the Care and Use of Laboratory Animals (NIH Publication No. 8023, revised 2011).

#### 2.3.2. Zebrafish 

Zebrafish were maintained at 28 °C under a 14 h (light) and 10 h (dark) cycle. Brine shrimps were raised in the laboratory and fed to the fish four times a day. To collect fertilized embryos, individual males and females were separated in a mating cage overnight and spawned the next morning. To evaluate toxicity to zebrafish embryos, fertilized eggs were washed five to six times with a solution of 4 ppm methylene blue in 0.1% egg water and washed again with a solution of 1 ppm methylene blue. Intact fertilized eggs selected under a microscope were plated in 96-well plates (5 eggs/well) in standard embryo water (1 g/L salt water with 100 μL/L Methylene Blue) and treated with various concentrations of FA for various times as indicated. Embryos were imaged using a DM2000 (Leica Co., Wetzlar, Germany) and a SZ2-ILST microscope (Olympus, Tokyo, Japan). All experiments on zebrafish were performed after obtaining approval by the Animal Care and Use Committee of Chungnam National University (CNU-00878).

### 2.4. Cell Viability Assay

Caco-2 cells were seeded on a 96-well plate and treated with various concentrations of FA for 24 h or 48 h. Viability were examined using an EZ-Cytox WST assay kit according to the instruction (Daeil Lab Service Co., Ltd., Seoul, Korea). Absorbances were measured at 450 nm using Microplate Spectrophotometer (xMark™, Bio-Rad, Hercules, CA, USA).

### 2.5. Nitric Oxide (NO) Assay

Polarized Caco-2 cells were pretreated apically with various concentrations of FA for 24 h and then apically with a cytokine cocktail for a further 24 h in the presence of FA. The amount of nitrite produced by cytokines in the upper chamber was measured using Griess reagent (Promega Co., Madison, WI, USA), according to the manufacturer’s instructions using a xMark™ Microplate Spectrophotometer. Amounts of NO produced were calculated using a NaNO_2_ reference plot.

### 2.6. Cell Permeability Assay

Polarized Caco-2 cells were treated with FA apically for 24 h then with the cytokine cocktail for 24 h. Upper and lower chambers were washed with HBSS/HEPES. FITC-dextran at 1 mg/mL was added to the upper chamber and fresh HBSS/HEPES was added to the lower chamber. Fluorescein absorbance from basal media in the lower chamber was measured over time using a multimode detector (DTX800, Beckman Coulter Inc., Brea, CA, USA).

### 2.7. Mouse Intestine Epithelial Cells (IECs) Isolation 

The mice were sacrificed, and the small intestine was immediately taken out and flushed with ice-cold PBS. For isolation of IECs, pieces of the small intestine were washed three times in ice-cold PBS and were incubated with 1 mM Dithiothreitol (Bio-Rad) for 10 min minutes in a shaker (Seiko Bioscience Co., Seongnam, Gyeonggi-do, Korea) in order to remove mucus in the lumen. After washing with PBS, pieces were incubated in 1 U/mL DISPASE II (Godo Shusei Co., Tokyo, Japan) at 37 °C for 30 min with shaker at 2500 rpm. The isolated epithelial cells were filtered through a 100 µm cell strainer (Becton, Dickinson and Co., Franklin lakes, NJ, USA) and centrifuged at 2500 rpm for 5 min. The cells were resuspended in 5 mL 100% Percoll (GE Healthcare, Chicago, IL, USA), followed by layering with 8 mL 40% Percoll in RPMI. Cells were centrifuged for 20 min at 1500 rpm without braking, and IECs were collected from the top 30% and washed with RPMI

### 2.8. Gene Expression in Real-Time Polymerase Chain Reaction (PCR)

Whole tissue (30 mg) was homogenized using a gentleMACS ™ Dissociator (Miltenyi Biotec. Co., Bergisch Gladbach, Germany) with TRI reagent (MRC Inc., Cincinnati, OH, USA) to isolate total RNA. For extraction from the cells, TRI reagent was added directly to the cells after removing the cell medium. Then, chloroform (Junsei Co., Tokyo, Japan) was added, and the homogenate was centrifuged at 12,000 rcf for 15 min at 4 °C. The supernatant was collected, isopropanol (Duksan Co., Ansan, Gyeonggi-do, Korea) was added, and centrifuged at 12,000 rcf for 8 min at 20 °C. The supernatant was removed, and the RNA concentration from the pellet was quantified using a NanoDrop^TM^ ONE spectrophotometers (NanoDrop ONE, Thermo Fisher Scientific Inc., Waltham, MA, USA). The cDNA synthesis was performed using an RT-Kit according to the instruction (M-MLV, RNase H-, Bio-Fact Co., Daejeon, Korea). mRNA expression levels were assessed by AriaMx1.0 Real Time PCR system (Agilent Co., Santa Clara, CA, USA) using the 2× Real-Time PCR Master Mix (Including SYBR Green I, Low ROX, Bio-Fact Co.). The primer sequences used are detailed in Table 1. 

### 2.9. Histological Staining of Intestines

Hematoxylin and Eosin (H&E) staining was performed to analyze the architecture of the intestine. The ileum section of the mouse was washed with 10% formaldehyde and stored until before analysis. From a paraffin block containing the samples, slices were cut, after mounted on glass slides, and stained with H&E (T&P Bio, Gwangju, Gyeonggi-do, Korea). All stained tissues were taken with an optical microscope (OLYM-PUS, Tokyo, Japan).

### 2.10. Intestinal Organoid Culture 

Intestines of male C57BL/6 mice littermates were opened longitudinally, washed with PBS. EDTA at 2.5 mM was added to small intestine pieces and shaken at 4 °C for 30 min. After settling down, basal medium (Advanced DMEM/F12) supplemented with Glu Max and HEPES was added to tissues, pipetted three times and centrifuged at 1200 rcf for 5 min. The pellets with organoid cells were resuspended in the same basal media and then passed through a 70 μm filter. The cell suspension was centrifuged down at 300 rcf for 2 min and the organoid pellets obtained were mixed with different concentration of FA or wild type cholera toxin as a positive control. The cell solution was mixed with Matrigel on a 24-well plate; once the Matrigel was polymerized, conditioned media supplemented with Wnt3a, R-spondin-3, and Noggin and basal media with B-27^®^ Supplement minus Vitamin A (B27), N-2 Supplement (N2), NAc (N-Acetyl-L-cysteine), Epidermal Growth Factor (EGF) were added to the gel. Organoids were imaged using a Leica stereomicroscope (Leica-Microsystems, Wetzlar, Germany).

### 2.11. Statistics

All experiments were conducted three or more times, and data analysis was conducted using SPSS/Windows 24.0 (SPSS Inc., Chicago, IL, USA). Representative results are presented as means ± SEM. A Student’s *t*-test was used to examine the difference between the mean values between the two groups. One-way ANOVA was performed to analyze the difference between the mean values of three or more groups. After one-way ANOVA, the difference between the independent variables was confirmed using Duncan’s multiple range test, and the statistical significance was defined as being statistically significant when *p* < 0.05.

## 3. Results

### 3.1. Determination of Ferulic Acid Concentration for Toxicity In Vitro and In Vivo

**Figure 1:**
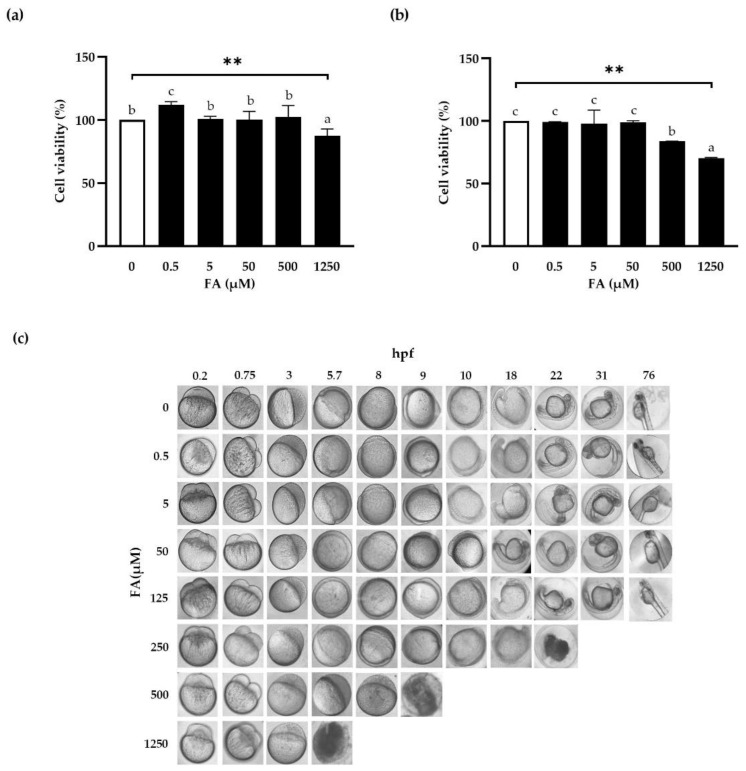
Effect of ferulic acid on cell viability. Caco-2 cells were treated with FA at the indicated concentrations for 24 h (**a**) or 48 h (**b**). Cell viabilities were measured using a WST assay. Results are presented as percentages of viable cells versus untreated cells. Zebrafish embryos were incubated with or without FA for 76 h per fertilization (hpf) (**c**). Pictures were taken at each time point. Data are the means ± SEM of three independent experiments. Duncan’s multiple range test was performed after one-way ANOVA to determine the significances of differences between FA groups. Significant differences are expressed as **: *p* < 0.01, and indicated by different letters. FA: ferulic acid; hpf: hours post fertilization.

To assess the dose-dependent toxicity of FA in vitro, cell viabilities were determined on Caco-2 using a WST assay (Figure 1). Up to 500 μM FA, cell survival was 100% for 24 h, while 1250 μM FA significantly reduced cell viability (Figure 1a). From a concentration of 500 μM, 48 h of treatment with FA drastically reduced cell viability (Figure 1b). Based on these results, FA was administered at a concentration of 0–500 μM for 24 h in subsequent experiments.

Although there is less than 70% gene homology between zebrafish and humans, zebrafish are commonly used as vertebrate animal models for drug toxicity, drug screening as well as the study of human diseases such as diabetes, obesity, fatty liver disease, and cardiovascular disease due to the high similarity between disease phenotypes [40,41,42]. Short assay times, reliability, straightforward maintenance, and environmental control are all advantages of these models, which require just modest amounts of drugs for quantitative assays. To optimize the concentration of FA used in subsequent experiments, we used a zebrafish embryo model for toxicity assessment. 

To investigate the toxicity of FA in vivo, zebrafish embryos were exposed to FA and their development was observed (Figure 1c). Embryos exposed to FA at concentration of 250 μM and higher failed to develop. When compared with untreated eggs, there was no overt phenotypic alteration (e.g., swelling of the cardiac cavity, digestive tract, or heart) and no circulation abnormalities, hyper-motility, growth stunting, or tail curvature once the eggs had hatched. 

### 3.2. FA Attenuated Nitric Oxide (NO) Production and Protected the Tight Junctions of Polarized Caco-2 Cells

**Figure 2:**
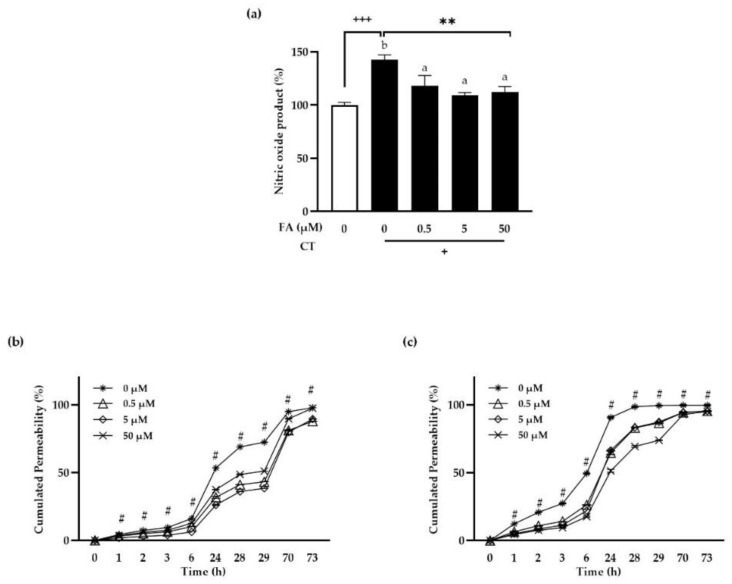
Ferulic acid’s antioxidant and protective effects on tight junctions in polarized Caco-2 cells. Polarized Caco-2 cells were pretreated with the indicated concentrations of FA apically for 24 h and then without (**b**) or with (**c**) cytokine cocktail (50 ng/mL human TNFα+ 50 ng/mL human IFNγ+ 25 ng/mL human IL1β+ 10 μg/mL LPS) apically for 24 h. Conditioned media were harvested, and NO levels were determined using Griess reagent (**a**). + means that CT is present. The cells were then washed and treated with HBSS buffer containing 1 mg/mL of FITC-dextran on apical sides. Fluorescein absorbance was measured at the indicated times. Data are the means ± SEM of the representative experiment. Duncan’s multiple range test was performed after one-way ANOVA to determine the significances of differences between FA groups. Significant differences are expressed as **: *p* < 0.01; #: *p* < 0.001, and indicated by different letters. The significances of stimulant effects in the absence of FA versus negative controls were determined using the Student’s *t*-test and are expressed as ^+++^: *p* < 0.001. FA: ferulic acid; CT: cytokine cocktail.

To investigate the anti-oxidation effect of FA, polarized intestinal epithelial cell lines, Caco-2 cells, were pretreated with FA for 24 h before being stimulated with CT for another 24 h. Griess reagents were then used to measure NO production (Figure 2a). CT increased NO levels significantly (*p* < 0.001) as compared to negative controls (unstimulated condition, white bar). However, CT-induced NO generation was significantly reduced even at the lowest concentration of FA, suggesting FA attenuates NO production induced by inflammation. 

To investigate how FA affects the tight junctions of polarized Caco-2 cells monolayer, the cells were pretreated with FA for 24 h and then stimulated with CT for another 24 h (Figure 2b,c, respectively). The cells were washed, and 1 mg/mL of FITC-dextran in HBSS buffer was introduced to the upper chamber. The fluorescence of the medium in the bottom chamber was measured at various time period and computed as percentage cumulated permeability versus 1 mg/mL of FITC-dextran. FA pretreatment significantly reduced permeability compared to untreated cells, implying that FA had a dose-dependent protective effect on epithelial cell tight junctions (Figure 2b). When cells pretreated with FA were stimulated with CT for 24 h to induce inflammation, cumulated permeability rate went up faster in 24 h compared to unstimulated condition and FA treated cells showed significantly reduced permeability than untreated controls (Figure 2c). These results suggest that FA preserve the tight junctions of intestinal epithelial cells in a dose response manner, regardless of the presence of inflammation.

### 3.3. FA Attenuated Pro-Inflammatory Response and ER Stress in Polarized Intestinal Epithelial Cells

**Figure 3:**
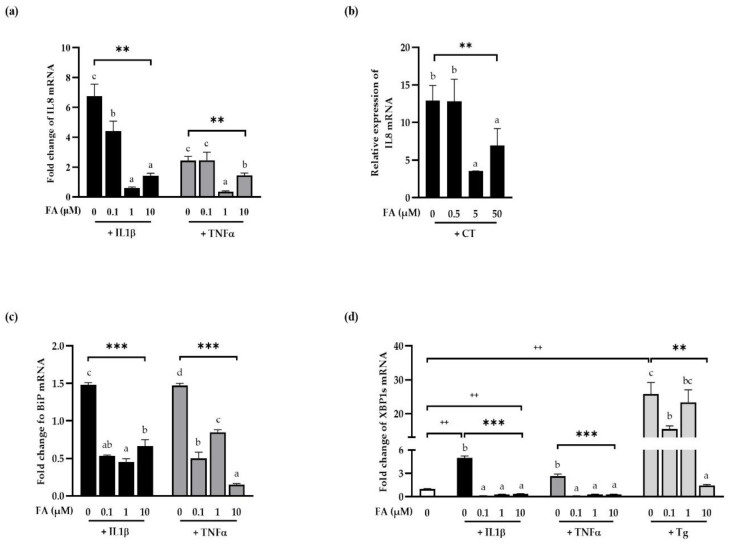
Inhibitory effect of ferulic acid on ER stress and proinflammatory response in polarized human intestinal epithelial cells. Polarized human intestinal epithelial cells ((**a**,**c**,**d**) T84 cells, (**b**) Caco-2 cells) were pretreated with the indicated concentrations of FA for 24 h and then treated with either 0.1 μg/mL IL1β only, 100 ng/mL TNFα only, or cytokine cocktail (50 ng/mL human TNFα+ 50 ng/mL human IFNγ+ 25 ng/mL human IL1β+ 10 μg/mL LPS) apically for 24 h. Three uM of Tg was treated apically for 1 h to examine XBP1s expression or 4 h to examine BiP expression. Total RNAs were extracted and cDNAs were prepared for real-time qPCR. Human IL8, BiP and XBP1s transcript levels were normalized versus human GAPDH. Data are the means ± SEM of the representative experiment. Duncan’s multiple range test was performed after one-way ANOVA to determine the significant differences between FA groups expressed as **: *p* < 0.01; ***: *p* < 0.001, and indicated by different letters. The significances of stimulant effects compared to negative controls in the absence of FA were determined using the Student’s *t*-test and are expressed as ^++^: *p* < 0.01. FA: ferulic acid; CT: cytokine cocktail; Tg: Thapsigargin.

We then examined pro-inflammatory cytokine levels in polarized human intestinal epithelial cells treated with FA to see how it affected the cells (Figure 3). The expression of IL8 mRNA, a pro-inflammatory response indicator, increased considerably when polarized T84 cells were treated with 0.1 μg/mL interleukin 1β (IL1β) or 100 ng/mL TNFα, (Figure 3a). FA, on the other hand, dramatically reduced IL8 mRNA expression induced by IL1β or TNFα, indicating that FA had an anti-inflammatory effect on human intestinal epithelial cells in the presence of inflammation. 

To rule out the possibility of various cell-specific phenotypes, we examined the effects of FA on Caco-2. As expected, IL8 induction was observed in Caco-2 cells treated with the cytokine cocktail (Figure 3b). FA at 5 and 50 μM attenuated this effect, although not in dose-dependent manner (*p* < 0.01). 

BiP is the hallmark of ER stress. We examined, then, whether cytokine-induced inflammation is related to ER stress by investigating BiP mRNA level in T84 cells treated with FA followed by IL1β or TNFα treatment (Figure 3c). As shown in Figure 3c, IL1β or TNFα treatment induced upregulation of BiP levels and this phenotype were inhibited by FA significantly, suggesting that FA might attenuate inflammation, which confirmed in above result, by downregulating ER stress. 

The spliced form of XBP1 (XBP1s) lay downstream of IRE1α activation, the most powerful ER stress sensor found on the ER membrane. Therefore, to confirm the effects of FA on ER stress in the presence of inflammation, the induction of XBP1s by FA was normalized and shown as fold changes (Figure 3d). Tg treatment was used as a positive control for ER stress induction as measured by XBP1s induction. The expression of XBP1s was significantly elevated by apical treatment with 0.1 μg/mL IL1β, 100 ng/mL TNFα or 3 uM Tg, showing that pro-inflammatory cytokines cause XBP1 splicing on human intestinal epithelial cells. Moreover, XBP1s levels induced by IL1β or TNFα were considerably reduced in the presence of FA, confirming FA prevented inflammation-induced ER stress.

In conclusion, FA attenuated both inflammation and ER stress induced by cytokines in polarized human intestinal epithelial T84 cells and Caco-2 cells, marking the first time FA has been shown to reduce inflammation and ER stress involving the cytokine-induced XBP1s pathway.

### 3.4. FA Attenuated Proinflammatory Response via IRE1a and PERK Pathways on Polarized Intestinal Epithelial Cells

**Figure 4:**
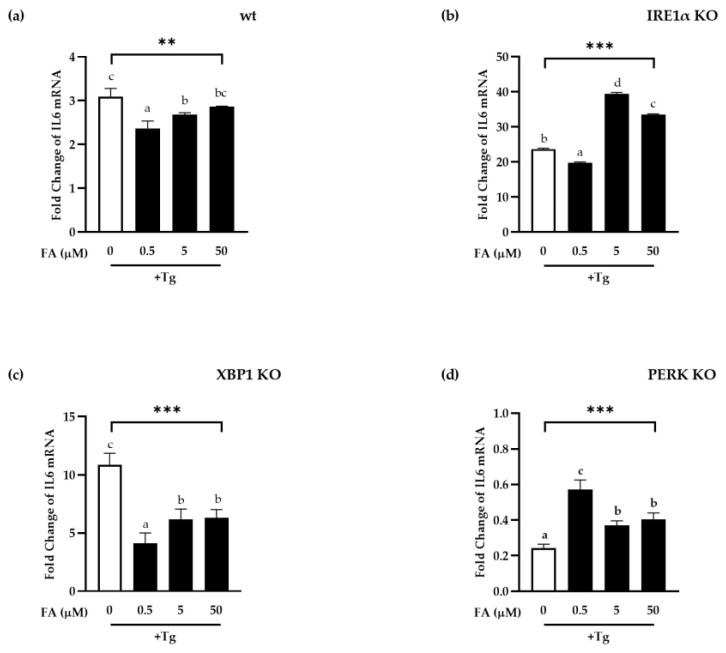
Effect of ferulic acid on the anti-inflammatory response in ER stress-induced MEF cells. FA were pretreated for 24 h before being treated for 4 h with Thapsigargin (Tg) on wild type (wt) (**a**), IRE1α Knock-Out (KO) (**b**), XBP1 KO (**c**), PERK KO (**d**) MEF cells. Total RNA were extracted and cDNA were prepared for real-time qPCR. Mouse IL6 and XBP1s level were normalized by mouse β-actin. Data are the means ± SEM of the representative experiment. Duncan’s multiple range test was performed after one-way ANOVA to determine the significant differences between FA groups, which were expressed as **: *p* < 0.01; ***: *p* < 0.001, and indicated by different letters. FA: ferulic acid; Tg: Thapsigargin.

The anti-inflammatory properties of FA were validated in the above experimental data, as was the reduction of ER stress caused by inflammation reflected by XBP1s. To identify the inhibitory mechanism of ER stress by FA, MEF cells were employed in which each gene regulating the ER stress pathway was knocked out (KO). The induction of IL6, a pro-inflammatory indicator, by FA itself was normalized and shown as fold changes (Figure 4).

Although 250 μM of FA greatly increased IL6 mRNA expression in wild type (wt) MEF cells in the unstimulated condition (data not shown), since it was proven that Tg treatment also relatively and significantly increased the pro-inflammatory index IL6, it resulted in a significant decrease in the fold change of IL6 mRNA level by FA (Figure 4a). 

Tg dramatically boosted IL6 induction in IRE1α KO cells lacking IRE1α, a major pathway of ER stress, most likely due to activation of alternate pathway via PERK or ATF6 (Figure 4b). However, Tg-induced IL6 mRNA expression was considerably higher in the presence of FA contrast to in wt MEF cells (Figure 4b). This implies that the IRE1α pathway is important for the inhibition of inflammation by FA.

In the presence of FA, however, XBP1 KO MEF cells with XBP1 gene deletion reduced inflammation, similar to the results of wt MEF cells, suggesting XBP1-independent IRE1α pathways in the mechanistic effect FA on ER stress (Figure 4c). On the other hand, Tg did not promote IL6 mRNA expression in PERK KO MEF cells in the absence of PERK (white bar, Figure 4d). Further research appears to be required, because the precise mechanism for this has yet to be determined. However, we can assume that PERK, rather than IRE1α, play a major role in Tg-induced IL6 induction at least in our MEF cell lines. Nevertheless, IL6 were induced by Tg significantly by FA in the absence of PERK, implying that PERK might play important role in the effect of FA inhibiting inflammation. 

As a result, our findings suggest that the XBP1-independent IRE1α and PERK pathways are involved in FA’s anti-inflammatory effect. Other ER stress indicators, such as BiP, PERK downstream target gene CHOP, IRE1α downstream target gene ERdj4, and EDM, will need to be identified in future investigation. 

### 3.5. FA Decreased Inflammatory Response of the Intestine In Vivo

**Figure 5:**
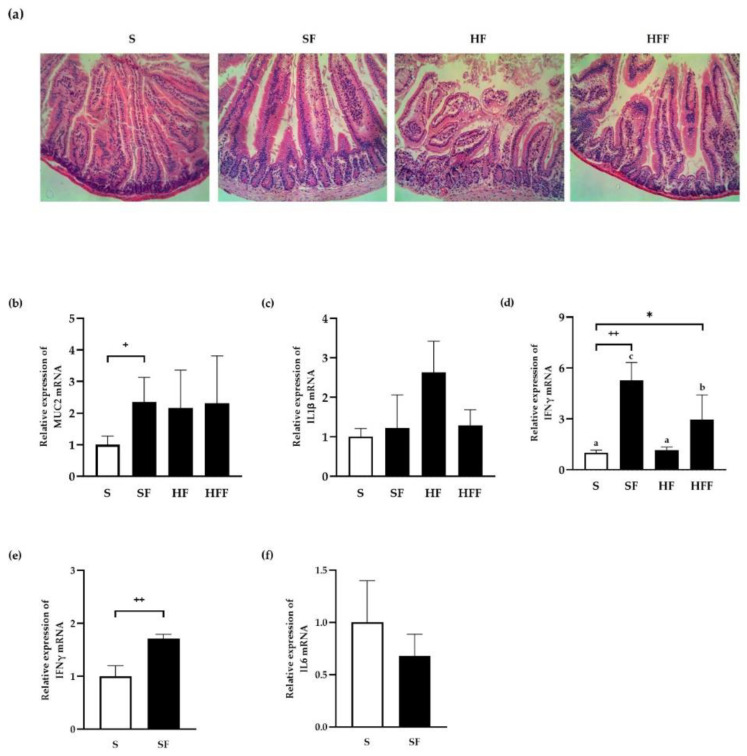
Anti-inflammatory effect of ferulic acid in vivo. The mice were fed a chow diet or a high-fat diet supplemented with 50 mg/mL FA for 8 weeks, and then whole intestinal tissues were harvested for H&E staining with 10× magnification (**a**) and qPCR measurements of Mucin 2 (**b**), IL1β (**c**), and IFNγ (**d**). Small intestine epithelial cells (IEC) were used to quantify IFNγ (**e**) and IL6 (**f**) by qPCR. Data are expressed as means ± SEM. Duncan’s multiple range test was performed after one-way ANOVA to determine the significances of group differences expressed as *: *p* < 0.05 and indicated by different letters. The significances of the effects of FA compared to control group were analyzed using the Student’s *t*-test ^+^: *p* < 0.05; ^++^: *p* < 0.01. S: standard chow diet; SF: standard chow diet supplemented with 50 mg/mL FA; HF: high-fat diet; HFF: high-fat diet supplemented with 50 mg/mL FA.

Obesity is caused by the accumulation of adipose tissue around peripheral blood vessels, which generates a hypoxic environment that promotes the secretion of inflammatory cytokine, leading to chronic mild inflammation [41]. For 8 weeks, mice were fed a high-fat diet (HFD) supplemented with 50 mg/mL FA to see how it works. The mice were then euthanized, their entire small intestines harvested, and intestinal epithelial cells (IECs) isolated (Figure 5).

Mice fed a regular chow diet supplemented with FA (SF) had similar intestines to animals fed a chow diet (S), whereas mice fed an HFD (HF) had greater lymphocyte infiltration than mice fed a chow diet (S) (Figure 5a). The villi of the small intestine were also disrupted in the HF group compared to SF or S groups. However, mice fed an HFD supplemented with FA (HFF) had less lymphocyte infiltration and epithelial barrier disruption than those observed in HF group.

To quantify inflammation, we measured the levels of proinflammatory markers in whole small intestine or IECs by real time PCR. Mucin 2 (oligomeric mucus gel forming protein), also known as MUC2, is a protein secreted onto mucosal surfaces by goblet cells in gut where it forms an insoluble mucous barrier that protects intestinal epithelium. Interestingly, we found MUC2 mRNA level on whole small intestines were significantly increased in mice fed with FA, which indicated FA somehow upregulates MUC2 mRNA expression to reinforce the protective mucin layer on intestinal epithelium (Figure 5b).

HFD-induced inflammation, as represented by IL1β induction, was reduced by FA supplementation, while the difference was not significant, supporting FA’s anti-inflammatory action in vitro (Figure 5c). 

IFNγ is a cytokine critical for both innate and adaptive immunity, an important activator of macrophages, and inducer of Class II major histocompatibility complex (MHC) molecule expression. The potential of IFNγ to inhibit viral replication directly, as well as its immunostimulatory and immunomodulatory effects, make it important in the immune system. Interestingly, even in the presence of mild inflammation by HFD, IFNγ was increased significantly by FA treatment on the entire small intestinal tissue (Figure 5d) as well as the IECs (Figure 5e) in the SF group compared to the S group. Since IFNγ is predominantly produced by natural killer (NK) and natural killer T (NKT) cells, our observation that FA makedly increased IFNγ expression in whole tissue comprising all other immune cells could indicate that FA plays an important role in host’s immune system (Figure 5d). FA also directly induced IFNγ in the intestinal epithelial cells, which serve as a direct defense barrier, as seen in Figure 5e, indicating that FA protected the immune system via an indirect immunomodulatory effect. 

Additionally, HFD did not induce IFNγ compared to control as expected and in the presence of mild inflammation represented as HFD, FA induced IFNγ mRNA level significantly in HFF group compared to HF group, confirming the immunomodulatory protective effect of FA.

In addition, albeit not significantly, proinflammatory cytokine IL6 mRNA expression was reduced in the IECs of mice fed with FA (Figure 5f). 

### 3.6. FA Increased Proliferation of the Mouse Small Intestine Organoids

**Figure 6:**
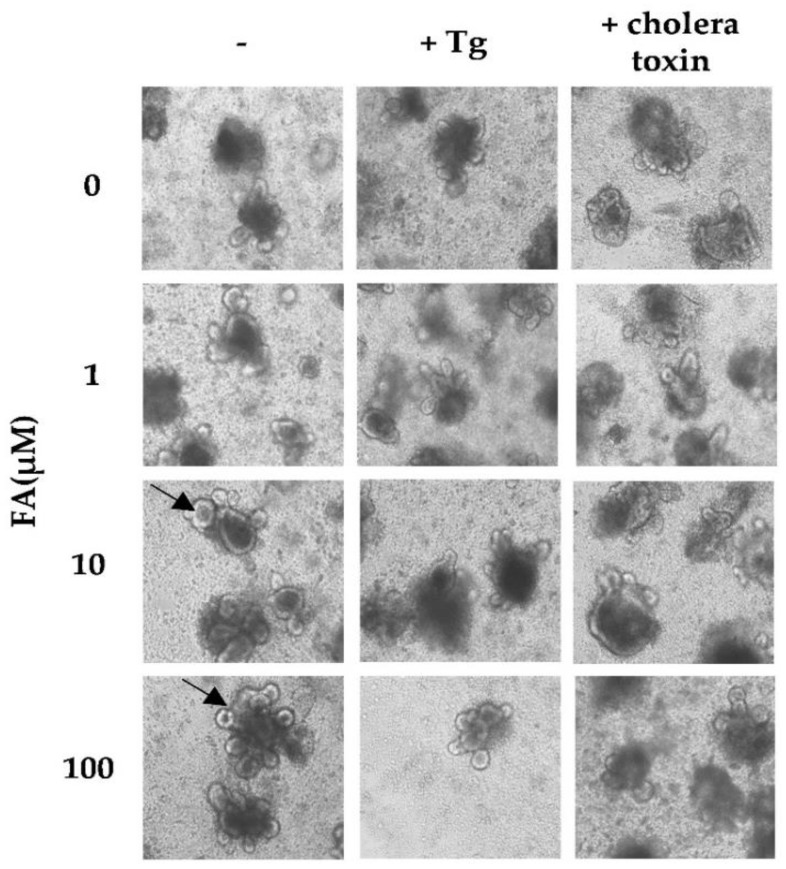
FA enhanced organoid proliferation of mouse small intestines. Small intestines were harvested, and organoids were isolated. Organoids were embedded into 3D Matrigel and cultured in the presence of FA for 1 week with or without 3 uM Tg or 3 nM cholera toxin. Pictures were taken after treatment with 40× magnification. Arrow shows the new proliferated organoid. Tg: Thapsigargin; FA: Ferulic acid.

Since FA was found to have anti-oxidative and anti-inflammatory effects and to protect the tight junctions of human intestinal epithelial cells, we used mouse intestinal organoids to investigate whether FA affects the normal development of intestinal stem cells. Small intestines were harvested, intestinal organoids were isolated and cultured in 3D Matrigel for a week in the absence or presence of FA to observe the proliferation of organoids. Cholera toxin, a representative of natural bacterial toxins that infect the intestine, was used as a positive control to examine whether the intestinal organoid system facilitated the absorption of FA into Matrigel properly. If the toxins functioned, organoids would die and turn black, and if they grew, they would show protruding blobs (black arrow). Organoids in our system showed a dose-dependent increase in protruding blobs with larger clumps in the presence of FA, suggesting that FA induces cell proliferation (Figure 6). However, treatment with 3 nM cholera toxin for 4 h incubation killed the organoids even in the presence of FA. In contrast, organoids did not proliferate in the presence of Tg when FA was absent. Organoids surprisingly proliferated when treated with FA in the presence of Tg, although the proliferation was comparatively reduced in the presence of FA. These results suggested that FA enhanced the proliferation and development of intestinal stem cell.

## 4. Discussion

Polyphenols are non-enzymatic antioxidants that provide cellular protection by neutralizing free radicals. FA is a phenolic acid abundant in plant cell walls, particularly those of vegetables and fruits; it has anti-oxidative and anti-inflammatory activities and protects by scavenging free radicals and other consequences of cell stress [1]. FA and their derivates have been identified in blood circulation and they are considered as the potential bioactive metabolites against inflammation [43]. 

The imbalance between the production of ROS and their elimination by antioxidant systems in the body is referred to as oxidative stress, and it can cause chronic inflammation. In many human pathological processes, persistent inflammatory states are prevalent. As pro-inflammatory mediators, cytokines promote a cell-mediated immune response and damage the intestinal epithelium. Moreover, intestinal inflammation results in the development of colitis if this stimulation persists [44,45]. Ulcerative Colitis (UC) and Crohn’s disease are forms of IBD, and the incidence of IBD in Asia has increased rapidly since the 1980s [46] and has been linked to environmental and dietary variables. Although the etiology and pathogenesis of IBD are uncertain, an imbalance in pro-inflammatory cytokines appears to play a role [47]. 

Recent research has connected a variety of UPR regulators to the inflammation and the etiology of IBD. Protein unfolding or misfolding in the ER of intestinal epithelial cells has been hypothesized to contribute directly to IBD, and IBD patients with active disease typically exhibit elevated levels of ER stress markers in the ileac and/or colonic epithelium [48,49]. Moreover, even unaffected tissues of IBD patients had higher ER and oxidative stress levels. Human genetic research has linked the UPR gene XBP1 to IBD, and XBP1 conditional knockout mice exhibited goblet cell deficit, mucin secretion failure, spontaneous inflammation in the small intestine, and impaired host defense against enterobacterial infection [37]. In addition, loss of XBP1 alleles increased JNK and NF-B pathways as well as the synthesis of inflammatory mediators in mucosa via significant activation of IRE1 in ileac epithelium. Therefore, in this study, we investigated the function of FA on gut health.

Previous studies have demonstrated that pretreatment with FA reduces NO accumulation in the culture medium of LPS-induced macrophage cells and has an inhibitory effect on the mRNA levels of various inflammatory mediators (e.g., IL6, TNFα, and iNOS) by inhibiting nuclear factor kappa B (NF-κB) activation and the Toll-like receptor (TLR) 4 pathway [50,51,52]. Furthermore, a clinical study revealed that FA administration ameliorated inflammation, concomitantly reduced plasma TNFα and IL6, and increased IL10 levels in human subjects [53,54]. Another study suggested that FA activated SIRT1 to protect the heart from the adverse effects of ER stress via reduction of PERK/eIF2α/ATF4/CHOP pathway [55].

In mice with a defective MUC2 gene, accumulating MUC2 precursor in the ER of goblet cells inhibited mucin production and damaged mucus layers. Mutant MUC2 mice also exhibited activation of innate and adaptive immunity mediated by the Th17 response in the colon, which is reminiscent of human UC [56]. Furthermore, the presence of misfolded MUC2 precursors in the ER of certain UC patients’ colonic goblet cells implies that protein folding defects are physiologically important to goblet cell pathology in the pathogenesis of UC. In MUC or IL10 mutant animals, IL10 (an anti-inflammatory cytokine essential for intestinal homeostasis) was also reported to reduce ER stress and promote mucin secretion by goblet cells [57,58]. Our mechanistic study confirmed that FA reduces NO production generated by cytokine stimulation, consistent with its antioxidant and anti-inflammatory effects on the intestinal epithelium in vitro and in vivo via XBP1-independent IRE1α and PERK pathways. In addition, interestingly enhancement of MUC2 was induced by FA in vivo. This implies that FA might be a supplementary tool for IBD treatment. 

The intestinal tract is lined with epithelial monolayer acting as a selective barrier for nutrients, electrolytes, and water while preventing the entry of intestinal pathogens, antigens, and toxins from the luminal environment to blood circulation and mesenteric lymph [59]. Therefore, tight junctions (TJs) of intestinal epithelial cells, composed of the transmembrane protein occludin and the cytoplasmic protein zonula occludens-1 (ZO-1) are key proteins that maintain TJs structure and intestinal epithelial barrier function, therefore important on paracellular permeability [60,61]. Dysfunction of intestinal epithelial barrier leads to increased permeability of intestinal mucosa, subsequent translocation of intestinal pathogenic bacteria or toxins, which in turn aggravates the damage of intestinal barrier integrity, resulting in local intestinal or systemic disease such as IBD and sepsis [62]. Therefore, maintenance of the intestinal epithelial barrier is very important in the clinical treatment of various acute and chronic diseases. 

Our conclusive findings indicate that FA protects the intestinal epithelial barrier against cellular stress and inflammation under both normal and stressful conditions. It has been reported that FA reduced the inflammation and alleviated intestinal barrier damage via upregulating TJ proteins in intestinal epithelial cells of rats and piglets [63,64]. One study showed the protective effect of FA on intestinal epithelial barrier in a lipopolysaccharide (LPS)-induced Caco-2 cells by increasing TJ proteins via PTEN/PI3K/AKT signaling pathway [65,66]. Another study used intestinal epithelial (IEC-6) cells and male Sprague–Dawley rats showed FA significantly attenuated the decrease in occludin, ZO-1 and E-cadherin expression by heat stress [67].

A very intriguing finding in our study is that in the presence of mild inflammation, IFNγ was increased significantly by FA on the small intestine in vivo. Potential mechanisms would be increasing histone deacetylase activity, regulating transcription factors, and activating TLR-7 pathway and it needs to be further investigated [68,69].

Due to the low absorption of polyphenol and phenolic metabolites, the biological activity of polyphenol seems to be important with gut microbiota. It is well-known that gut microbiota-derived metabolic substances like short-chain fatty acids (SCFA) can promote gut immunity [70], whereas gut microbiota disturbance can induce intestinal inflammation and barrier damage [71]. In a human study, FA intake was shown to enhance the number of Bacteroidetes and decrease the population of Firmicutes in the microbiome analysis [72]. In vivo studies have also shown that FA can mitigate intestinal inflammation, promote the growth of Bacteroides, and induce the production of SCFAs by modulating the gut microbiota in mouse and diabetic syndrome rat model [73,74]. Feruloyl Oligosaccharides (FOs), the ferulic acid ester of oligosaccharides, has been reported to strengthen the antioxidative capacity of the jejunum and alter the structure and composition of the cecal microbiota in rats by increasing the relative abundances of Actinobacteria, Proteobacteria while decreasing the abundance of Firmicutes. [75]. In the future, it would be interesting to follow the gut microbiome-modulating effect of FA on gut health. 

Although zebrafish embryos are increasingly used for developmental toxicity screening of candidate drugs for their advantage, and that is the reason we used zebrafish model for in vivo toxicity of FA, keep in mind that false negative and positive results were reported even from the same compound between laboratories [76,77,78]. The reasons are thought to be due to the large diversity in protocols and species difference, indicating a clear need for harmonization of the zebrafish assays in the future.

In addition, FA has been shown to enhance the differentiations of various stem cells (e.g., neuronal and skeletal stem cells) by activating the p38/mitogen-activated protein kinase (MAPK) and extracellular signal-regulated kinase (ERK)/MAPK pathways, and for this reason, some have used FA in stem cell therapies [22,79,80]. In HFD-induced obese mice, FA has also been shown to retain the self-renewal capacity of embryo stem cells and adipose-derived mesenchymal stem cells [81,82]. Surprisingly, it is a notable finding that in our system FA also boosted the proliferation of adult intestinal stem cells, which is important for the 3–4-day intestinal epithelial regeneration that occurs in the human body. Therefore, in the present study, we examined the protective effect of FA against inflammatory responses in human intestinal epithelial cells. Furthermore, our results show that FA ameliorated proinflammatory response induced by proinflammatory cytokines, implying that FA might improve gut health and protects against external and endogenous inflammation as a result. 

## 5. Conclusions

Our data demonstrate that FA promotes the survival and differentiation of adult small intestinal stem cells, prevents inflammation, and contributes to the maintenance of gut health, hence supporting its therapeutic potential. Moreover, the study shows FA protects the intestinal barrier. Therefore, we propose that FA be considered as a potential preventive or alleviating component in the diet of IBD patients. 

## Figures and Tables

**Table 1:** Real-time PCR primer sequences for human and mouse.

Gene	Accession Number	Primer	Sequence (5′→3′)
hGAPDH	NM_001357943.2	Fw	ATG GGG AAG GTG AAG GTG G
		Rv	GGG GTG ATT GAT GGC AAC AAT A
hIL8	NM_001354840.3	Fw	GTT TTT GAA GAG GGC TGA GAA TTC
		Rv	CAT GAA GTG TTG AAG TAG ATT TGC TTG
hXBP1s	NM_001393999.1	Fw	AAC CAG GAG TTA AGA CAG CGC TT
		Rv	CTG CAC CCT CTG CGG ACT
mβ-actin	NM_007393.5	Fw	GGC TGT ATT CCC CTC CAT CG
		Rv	CCA CTT GGT AAC AAT GCC ATG T
mMUC2	NM_023566.4	Fw	GCC TGT TTG ATA GCT GCT ATG TGC C
		Rv	GTT CCG CCA GTC AAT GCA GAC AC
mIL1β	NM_008361.4	Fw	GAA ATG CCA CCT TTT GAC AGT G
		Rv	TGG ATG CTC TCA TCA GGA CAG
mIL10	NM_010548.2	Fw	CTT ACT GAC TGG CAT GAG GAT CA
		Rv	GCA GCT CTA GGA GCA TGT GG
mIFNγ	NM_008337.4	Fw	TCA AGT GGC ATA GAT GTG GAA GAA
		Rv	TGG CTC TGC AGG ATT TTC ATG
mIL6	NM_001314054.1	Fw	CTG CAA GAG ACT TCC ATC CAG
		Rv	AGT GGT ATA GAC AGG TCT GTT GG

## Data Availability

Data is contained within the article.

## References

[1] Zhao Z., Moghadasian M.H. (2008). Chemistry, natural sources, dietary intake and pharmacokinetic properties of ferulic acid: A review. Food Chem..

[2] Adom K.K., Sorrells M.E., Liu R.H. (2003). Phytochemical profiles and antioxidant activity of wheat varieties. J. Agric. Food Chem..

[3] Andreasen M.F., Christensen L.P., Meyer A.S., Hansen A. (2000). Content of phenolic acids and ferulic acid dehydrodimers in 17 rye (*Secale cereale* L.) varieties. J. Agric. Food Chem..

[4] Sun R.C., Sun X.F., Zhang S.H. (2001). Quantitative determination of hydroxycinnamic acids in wheat, rice, rye, and barley straws, maize stems, oil palm frond fiber, and fast-growing poplar wood. J. Agric. Food Chem..

[5] Hartley R.D., Harris P.J. (1981). Phenolic Constituents of the cell walls of dicotyledons. Biochem. Syst. Ecol..

[6] Harris P.J., Hartley R.D. (1980). Phenolic constituents of the cell walls of monocotyledons. Biochem. Syst. Ecol..

[7] Ergun B.C., Coban T., Onurdag F.K., Banoglu E. (2011). Synthesis, antioxidant and antimicrobial evaluation of simple aromatic esters of ferulic acid. Arch. Pharm. Res..

[8] Srinivasan M., Sudheer A.R., Menon V.P. (2007). Ferulic acid: Therapeutic potential through its antioxidant property. J. Clin. Biochem. Nutr..

[9] Chowdhury S., Ghosh S., Rashid K., Sil P.C. (2016). Deciphering the role of ferulic acid against streptozotocin-induced cellular stress in the cardiac tissue of diabetic rats. Food Chem. Toxicol..

[10] Lee H.C., Jenner A.M., Low C.S., Lee Y.K. (2006). Effect of tea phenolics and their aromatic fecal bacterial metabolites on intestinal microbiota. Res. Microbiol..

[11] Yan J.J., Cho J.Y., Kim H.S., Kim K.L., Jung J.S., Huh S.O., Suh H.W., Kim Y.H., Song D.K. (2001). Protection against beta-amyloid peptide toxicity in vivo with long-term administration of ferulic acid. Br. J. Pharmacol..

[12] Turkez H., Arslan M.E., Barboza J.N., Kahraman C.Y., de Sousa D.P., Mardinoglu A. (2022). Therapeutic Potential of Ferulic Acid in Alzheimer’s Disease. Curr. Drug Deliv..

[13] Wang N.Y., Li J.N., Liu W.L., Huang Q., Li W.X., Tan Y.H., Liu F., Song Z.H., Wang M.Y., Xie N. (2021). Ferulic Acid Ameliorates Alzheimer’s Disease-like Pathology and Repairs Cognitive Decline by Preventing Capillary Hypofunction in APP/PS1 Mice. Neurotherapeutics.

[14] Wang E.J., Wu M.Y., Lu J.H. (2021). Ferulic Acid in Animal Models of Alzheimer’s Disease: A Systematic Review of Preclinical Studies. Cells.

[15] Singh Y.P., Rai H., Singh G., Singh G.K., Mishra S., Kumar S., Srikrishna S., Modi G. (2021). A review on ferulic acid and analogs based scaffolds for the management of Alzheimer’s disease. Eur. J. Med. Chem..

[16] Salamanova E., Atanasova M., Dimitrov I., Doytchinova I. (2021). Effects of Curcumin and Ferulic Acid on the Folding of Amyloid-beta Peptide. Molecules.

[17] Pueknang J., Saewan N. (2022). Stability and Anti-Aging of Encapsulated Ferulic Acid in Phosphorylated Rice Starch. Molecules.

[18] Chang C.J., Chiu J.H., Tseng L.M., Chang C.H., Chien T.M., Wu C.W., Lui W.Y. (2006). Modulation of HER2 expression by ferulic acid on human breast cancer MCF7 cells. Eur. J. Clin. Investig..

[19] Fong Y., Tang C.C., Hu H.T., Fang H.Y., Chen B.H., Wu C.Y., Yuan S.S., Wang H.D., Chen Y.C., Teng Y.N. (2016). Inhibitory effect of trans-ferulic acid on proliferation and migration of human lung cancer cells accompanied with increased endogenous reactive oxygen species and beta-catenin instability. Chin. Med..

[20] Janicke B., Hegardt C., Krogh M., Onning G., Akesson B., Cirenajwis H.M., Oredsson S.M. (2011). The antiproliferative effect of dietary fiber phenolic compounds ferulic acid and p-coumaric acid on the cell cycle of Caco-2 cells. Nutr. Cancer.

[21] Lin C.M., Chiu J.H., Wu I.H., Wang B.W., Pan C.M., Chen Y.H. (2010). Ferulic acid augments angiogenesis via VEGF, PDGF and HIF-1 alpha. J. Nutr. Biochem..

[22] Gu L., Cui X., Wei W., Yang J., Li X. (2017). Ferulic acid promotes survival and differentiation of neural stem cells to prevent gentamicin-induced neuronal hearing loss. Exp. Cell Res..

[23] Yabe T., Hirahara H., Harada N., Ito N., Nagai T., Sanagi T., Yamada H. (2010). Ferulic acid induces neural progenitor cell proliferation in vitro and in vivo. Neuroscience.

[24] Doss H.M., Dey C., Sudandiradoss C., Rasool M.K. (2016). Targeting inflammatory mediators with ferulic acid, a dietary polyphenol, for the suppression of monosodium urate crystal-induced inflammation in rats. Life Sci..

[25] Hiratsuka T., Matsuzaki S., Miyata S., Kinoshita M., Kakehi K., Nishida S., Katayama T., Tohyama M. (2010). Yokukansan inhibits neuronal death during ER stress by regulating the unfolded protein response. PLoS ONE.

[26] Hotamisligil G.S., Davis R.J. (2016). Cell Signaling and Stress Responses. Cold Spring Harb. Perspect. Biol..

[27] Ron D., Walter P. (2007). Signal integration in the endoplasmic reticulum unfolded protein response. Nat. Rev. Mol. Cell Biol..

[28] Walter P., Ron D. (2011). The unfolded protein response: From stress pathway to homeostatic regulation. Science.

[29] Ron D., Hubbard S.R. (2008). How IRE1 reacts to ER stress. Cell.

[30] Sun S., Shi G., Sha H., Ji Y., Han X., Shu X., Ma H., Inoue T., Gao B., Kim H. (2015). IRE1alpha is an endogenous substrate of endoplasmic-reticulum-associated degradation. Nat. Cell Biol..

[31] Lee A.H., Iwakoshi N.N., Glimcher L.H. (2003). XBP-1 regulates a subset of endoplasmic reticulum resident chaperone genes in the unfolded protein response. Mol. Cell Biol..

[32] Kaufman R.J. (1999). Stress signaling from the lumen of the endoplasmic reticulum: Coordination of gene transcriptional and translational controls. Genes Dev..

[33] Harding H.P., Novoa I., Zhang Y., Zeng H., Wek R., Schapira M., Ron D. (2000). Regulated translation initiation controls stress-induced gene expression in mammalian cells. Mol. Cell.

[34] Szegezdi E., Logue S.E., Gorman A.M., Samali A. (2006). Mediators of endoplasmic reticulum stress-induced apoptosis. EMBO Rep..

[35] Kaser A., Blumberg R.S. (2009). Endoplasmic reticulum stress in the intestinal epithelium and inflammatory bowel disease. Semin. Immunol..

[36] Kaser A., Blumberg R.S. (2010). Survive an innate immune response through XBP1. Cell Res..

[37] Kaser A., Lee A.H., Franke A., Glickman J.N., Zeissig S., Tilg H., Nieuwenhuis E.E., Higgins D.E., Schreiber S., Glimcher L.H. (2008). XBP1 links ER stress to intestinal inflammation and confers genetic risk for human inflammatory bowel disease. Cell.

[38] Kaser A., Martinez-Naves E., Blumberg R.S. (2010). Endoplasmic reticulum stress: Implications for inflammatory bowel disease pathogenesis. Curr. Opin Gastroenterol..

[39] Hotamisligil G.S. (2010). Endoplasmic reticulum stress and the inflammatory basis of metabolic disease. Cell.

[40] Santoriello C., Zon L.I. (2012). Hooked! Modeling human disease in zebrafish. J. Clin. Investig..

[41] Langheinrich U. (2003). Zebrafish: A new model on the pharmaceutical catwalk. Bioessays.

[42] Dooley K., Zon L.I. (2000). Zebrafish: A model system for the study of human disease. Curr. Opin. Genet. Dev..

[43] de Ferrars R.M., Cassidy A., Curtis P., Kay C.D. (2014). Phenolic metabolites of anthocyanins following a dietary intervention study in post-menopausal women. Mol. Nutr. Food Res..

[44] Singh U.P., Singh N.P., Murphy E.A., Price R.L., Fayad R., Nagarkatti M., Nagarkatti P.S. (2016). Chemokine and cytokine levels in inflammatory bowel disease patients. Cytokine.

[45] Hoang P., Fiasse R., Van Heuverzwyn R., Sibille C. (1994). Role of cytokines in inflammatory bowel disease. Acta Gastroenterol. Belg..

[46] Prideaux L., Kamm M.A., De Cruz P.P., Chan F.K., Ng S.C. (2012). Inflammatory bowel disease in Asia: A systematic review. J. Gastroenterol. Hepatol..

[47] Ardizzone S., Bianchi Porro G. (2005). Biologic therapy for inflammatory bowel disease. Drugs.

[48] Camarillo G.F., Goyon E.I., Zuniga R.B., Salas L.A.S., Escarcega A.E.P., Yamamoto-Furusho J.K. (2020). Gene Expression Profiling of Mediators Associated with the Inflammatory Pathways in the Intestinal Tissue from Patients with Ulcerative Colitis. Mediat. Inflamm..

[49] Coope A., Pascoal L.B., Botezelli J.D., da Silva F.A.R., Ayrizono M.L.S., Rodrigues B.L., Milanski M., Carvalho R.B., Fagundes J.J., Velloso L.A. (2019). ER stress activation in the intestinal mucosa but not in mesenteric adipose tissue is associated with inflammation in Crohn’s disease patients. PLoS ONE.

[50] Lampiasi N., Montana G. (2016). The molecular events behind ferulic acid mediated modulation of IL-6 expression in LPS-activated Raw 264.7 cells. Immunobiology.

[51] Yuan J., Ge K., Mu J., Rong J., Zhang L., Wang B., Wan J., Xia G. (2016). Ferulic acid attenuated acetaminophen-induced hepatotoxicity though down-regulating the cytochrome P 2E1 and inhibiting toll-like receptor 4 signaling-mediated inflammation in mice. Am. J. Transl. Res..

[52] Yin P., Zhang Z., Li J., Shi Y., Jin N., Zou W., Gao Q., Wang W., Liu F. (2019). Ferulic acid inhibits bovine endometrial epithelial cells against LPS-induced inflammation via suppressing NK-kappaB and MAPK pathway. Res. Vet. Sci..

[53] Katcher H.I., Legro R.S., Kunselman A.R., Gillies P.J., Demers L.M., Bagshaw D.M., Kris-Etherton P.M. (2008). The effects of a whole grain-enriched hypocaloric diet on cardiovascular disease risk factors in men and women with metabolic syndrome. Am. J. Clin. Nutr..

[54] Vitaglione P., Mennella I., Ferracane R., Rivellese A.A., Giacco R., Ercolini D., Gibbons S.M., La Storia A., Gilbert J.A., Jonnalagadda S. (2015). Whole-grain wheat consumption reduces inflammation in a randomized controlled trial on overweight and obese subjects with unhealthy dietary and lifestyle behaviors: Role of polyphenols bound to cereal dietary fiber. Am. J. Clin. Nutr..

[55] Monceaux K., Gressette M., Karoui A., Pires Da Silva J., Piquereau J., Ventura-Clapier R., Garnier A., Mericskay M., Lemaire C. (2022). Ferulic Acid, Pterostilbene, and Tyrosol Protect the Heart from ER-Stress-Induced Injury by Activating SIRT1-Dependent Deacetylation of eIF2alpha. Int. J. Mol. Sci..

[56] Eri R.D., Adams R.J., Tran T.V., Tong H., Das I., Roche D.K., Oancea I., Png C.W., Jeffery P.L., Radford-Smith G.L. (2011). An intestinal epithelial defect conferring ER stress results in inflammation involving both innate and adaptive immunity. Mucosal. Immunol..

[57] Burger-van Paassen N., van der Sluis M., Bouma J., Korteland-van Male A.M., Lu P., Van Seuningen I., Boehm G., van Goudoever J.B., Renes I.B. (2011). Colitis development during the suckling-weaning transition in mucin Muc2-deficient mice. Am. J. Physiol. Gastrointest. Liver Physiol..

[58] Schwerbrock N.M., Makkink M.K., van der Sluis M., Buller H.A., Einerhand A.W., Sartor R.B., Dekker J. (2004). Interleukin 10-deficient mice exhibit defective colonic Muc2 synthesis before and after induction of colitis by commensal bacteria. Inflamm. Bowel. Dis..

[59] Odenwald M.A., Turner J.R. (2017). The intestinal epithelial barrier: A therapeutic target?. Nat. Rev. Gastroenterol. Hepatol..

[60] Buckley A., Turner J.R. (2018). Cell Biology of Tight Junction Barrier Regulation and Mucosal Disease. Cold Spring Harb. Perspect. Biol..

[61] Costantini T.W., Deree J., Loomis W., Putnam J.G., Choi S., Baird A., Eliceiri B.P., Bansal V., Coimbra R. (2009). Phosphodiesterase inhibition attenuates alterations to the tight junction proteins occludin and ZO-1 in immunostimulated Caco-2 intestinal monolayers. Life Sci..

[62] Michielan A., D’Inca R. (2015). Intestinal Permeability in Inflammatory Bowel Disease: Pathogenesis, Clinical Evaluation, and Therapy of Leaky Gut. Mediat. Inflamm..

[63] Wang X., Yang F., Na L., Jia M., Ishfaq M., Zhang Y., Liu M., Wu C. (2022). Ferulic acid alleviates AFB1-induced duodenal barrier damage in rats via up-regulating tight junction proteins, down-regulating ROCK, competing CYP450 enzyme and activating GST. Ecotoxicol. Environ. Saf..

[64] Hu R., Wu S., Li B., Tan J., Yan J., Wang Y., Tang Z., Liu M., Fu C., Zhang H. (2022). Dietary ferulic acid and vanillic acid on inflammation, gut barrier function and growth performance in lipopolysaccharide-challenged piglets. Anim. Nutr..

[65] He S., Guo Y., Zhao J., Xu X., Wang N., Liu Q. (2020). Ferulic Acid Ameliorates Lipopolysaccharide-Induced Barrier Dysfunction via MicroRNA-200c-3p-Mediated Activation of PI3K/AKT Pathway in Caco-2 Cells. Front. Pharmacol..

[66] Kim H.J., Lee E.K., Park M.H., Ha Y.M., Jung K.J., Kim M.S., Kim M.K., Yu B.P., Chung H.Y. (2013). Ferulate protects the epithelial barrier by maintaining tight junction protein expression and preventing apoptosis in tert-butyl hydroperoxide-induced Caco-2 cells. Phytother. Res..

[67] He S., Liu F., Xu L., Yin P., Li D., Mei C., Jiang L., Ma Y., Xu J. (2016). Protective Effects of Ferulic Acid against Heat Stress-Induced Intestinal Epithelial Barrier Dysfunction In Vitro and In Vivo. PLoS ONE.

[68] Ferreira A.O., Polonini H.C., Dijkers E.C.F. (2020). Postulated Adjuvant Therapeutic Strategies for COVID-19. J. Pers. Med..

[69] Ma Z.C., Hong Q., Wang Y.G., Liang Q.D., Tan H.L., Xiao C.R., Tang X.L., Shao S., Zhou S.S., Gao Y. (2011). Ferulic acid induces heme oxygenase-1 via activation of ERK and Nrf2. Drug Discov. Ther..

[70] Donia M.S., Fischbach M.A. (2015). HUMAN MICROBIOTA. Small molecules from the human microbiota. Science.

[71] Guevarra R.B., Hong S.H., Cho J.H., Kim B.R., Shin J., Lee J.H., Kang B.N., Kim Y.H., Wattanaphansak S., Isaacson R.E. (2018). The dynamics of the piglet gut microbiome during the weaning transition in association with health and nutrition. J. Anim. Sci. Biotechnol..

[72] Martinez I., Lattimer J.M., Hubach K.L., Case J.A., Yang J., Weber C.G., Louk J.A., Rose D.J., Kyureghian G., Peterson D.A. (2013). Gut microbiome composition is linked to whole grain-induced immunological improvements. ISME J..

[73] Tian B., Geng Y., Wang P., Cai M., Neng J., Hu J., Xia D., Cao W., Yang K., Sun P. (2022). Ferulic acid improves intestinal barrier function through altering gut microbiota composition in high-fat diet-induced mice. Eur. J. Nutr..

[74] Song Y., Wu M.S., Tao G., Lu M.W., Lin J., Huang J.Q. (2020). Feruloylated oligosaccharides and ferulic acid alter gut microbiome to alleviate diabetic syndrome. Food Res. Int..

[75] Wang W., Wang Y., Duan Y., Meng Z., An X., Qi J. (2022). Regulation of wheat bran feruloyl oligosaccharides in the intestinal antioxidative capacity of rats associated with the p38/JNK-Nrf2 signaling pathway and gut microbiota. J. Sci. Food Agric..

[76] Gustafson A.L., Stedman D.B., Ball J., Hillegass J.M., Flood A., Zhang C.X., Panzica-Kelly J., Cao J., Coburn A., Enright B.P. (2012). Inter-laboratory assessment of a harmonized zebrafish developmental toxicology assay—Progress report on phase I. Reprod. Toxicol..

[77] Ball J.S., Stedman D.B., Hillegass J.M., Zhang C.X., Panzica-Kelly J., Coburn A., Enright B.P., Tornesi B., Amouzadeh H.R., Hetheridge M. (2014). Fishing for teratogens: A consortium effort for a harmonized zebrafish developmental toxicology assay. Toxicol. Sci..

[78] Teixido E., Pique E., Gomez-Catalan J., Llobet J.M. (2013). Assessment of developmental delay in the zebrafish embryo teratogenicity assay. Toxicol. Vitr..

[79] Liang J.W., Li P.L., Wang Q., Liao S., Hu W., Zhao Z.D., Li Z.L., Yin B.F., Mao N., Ding L. (2021). Ferulic acid promotes bone defect repair after radiation by maintaining the stemness of skeletal stem cells. Stem. Cells Transl. Med..

[80] Moghadam F.H., Mesbah-Ardakani M., Nasr-Esfahani M.H. (2018). Ferulic Acid exerts concentration-dependent anti-apoptotic and neuronal differentiation-inducing effects in PC12 and mouse neural stem cells. Eur. J. Pharmacol..

[81] Cho J., Park E. (2020). Ferulic acid maintains the self-renewal capacity of embryo stem cells and adipose-derived mesenchymal stem cells in high fat diet-induced obese mice. J. Nutr. Biochem..

[82] Lee H., Cho J.A., Park E. (2020). Cell cycle profile data on splenocytes of high fat diet induced obese mice treated with ferulic acid. Data Brief.

